# Effects of the Small-Sided Soccer Games on Blood Pressure in Untrained Hypertensive Adults: A Systematic Review and Meta-Analysis of Randomized Controlled Trials

**DOI:** 10.3390/healthcare9030345

**Published:** 2021-03-18

**Authors:** Filipe Manuel Clemente, Rodrigo Ramirez-Campillo, Hugo Sarmento

**Affiliations:** 1 Escola Superior Desporto e Lazer, Instituto Politécnico de Viana do Castelo, Rua Escola Industrial e Comercial de Nun’Álvares, 4900-347 Viana do Castelo, Portugal; 2 Instituto de Telecomunicações, Delegação da Covilhã, 1049-001 Lisboa, Portugal; 3 Human Performance Laboratory, Department of Physical Activity Sciences, Universidad de Los Lagos, Lord Cochrane 1046, Osorno 5290000, Chile; r.ramirez@ulagos.cl; 4 Centro de Investigación en Fisiología del Ejercicio, Facultad de Ciencias, Universidad Mayor, Santiago 7500000, Chile; 5 Research Unit for Sport and Physical Activity, Faculty of Sport Sciences and Physical Education, University of Coimbra, 3004-531 Coimbra, Portugal; hg.sarmento@gmail.com

**Keywords:** football, hypertension, non-communicable diseases, recreational football, health promotion

## Abstract

This systematic review with meta-analysis was conducted to assess the effects of small-sided games (SSGs)-based programs on the systolic and diastolic blood pressure of untrained hypertensive adults. The data sources utilized were Web of Science, Scopus, SPORTDiscus, and PubMed. The eligibility criteria were: (i) randomized controlled trials including a control group and an intervention group exclusively using soccer SSGs; (ii) intervention and control groups including an untrained hypertensive adult population; (iii) articles written in English; and (iv) only full-text and original articles. The database search initially identified 241 titles. From those, five articles were eligible for the systematic review and meta-analysis. The included randomized controlled studies involved five individual experimental groups and 88 participants, and 68 participants in the five control groups. The results showed a large and beneficial effect of SSG on systolic (ES = 1.69; 95% CI = 0.71 to 2.66; *p =* 0.001; *I*^2^ = 85.2%; Egger’s test *p* = 0.101) and diastolic blood pressure (ES = 2.25; 95% CI = 1.44 to 3.06; *p <* 0.001; *I*^2^ = 74.8%; Egger’s test *p* = 0.118) when compared to the control groups. The findings of the current systematic review and meta-analysis revealed consistent beneficial effects of recreational soccer SSGs on untrained men and women from the hypertensive population, although high levels of heterogeneity.

## 1. Introduction

Arterial hypertension can be considered a chronic elevation of resting arterial blood pressure [1]. The level of hypertension severity can differ considering the values registered and can be classified as stage 1 (>140/90 mm Hg systolic/diastolic blood pressure), stage 2 (>160/100 mm Hg systolic/diastolic blood pressure), or stage 3 (>180/110 mm Hg systolic/diastolic blood pressure) [2]. Usually, hypertension is diagnosed after multiple measurements on at least two days separated by an interval of 1–4 weeks.

Worldwide, ~41% of the population aged between 35 and 70 years old has hypertension [3]. Despite being generally asymptomatic, hypertension (or high blood pressure) significantly increases the risk of death, cardiovascular mortality, and cardiovascular disease (e.g., stroke, heart failure, coronary artery disease) [4]. For that reason, clinical diagnoses and subsequent pharmacological [5] and non-pharmacological [6] approaches to control the condition help reduce the risk of complications.

There are a list of determinant approaches to moderate the risk of increasing hypertension [7]: (a) physical activity/exercise; (b) controlling or reducing the body mass (or weight loss) or reducing the body fat; (c) moderation of alcohol and caffeine consumption; (d) moderation of salt (sodium) consumption; and (e) adoption of Dietary Approaches to Stop Hypertension (DASH). Among the non-pharmacological approaches to treating hypertension, physical exercise is a determinant factor. A meta-analysis summarizing 54 clinical trials revealed that aerobic exercise reduced 3.85 mm Hg and 2.58 mm Hg in systolic and diastolic blood pressure, respectively [8]. In a more recent meta-analysis that included 59 randomized clinical trials (of endurance exercise), it was also found that blood pressure reduced to a greater extent after endurance training performed at a moderate to high intensity (−3.5 and −2.5 mm Hg in systolic and diastolic blood pressure, respectively) [1].

Endurance training seems to be the most effective form of exercise among the remaining training options for lowering blood pressure [1,9,10]. However, endurance training can be prescribed in different ways, and this should be considered when motivating and engaging the hypertensive population to regularly practice physical exercise [9]. Following this idea, researchers have tested recreational soccer as a way to encourage people to live healthy lifestyles, increase their level of physical exercise, and improve the general health of the clinical population [10,11].

Soccer practice can be promoted in different ways. The regular format of play (11 vs. 11) is one way to implement exercise into people’s lives. However, the regular format of play (11 vs. 11) represents a moderate physiological level in participants since there is a smaller number of individual participations regarding the ball, thus the match becomes more positional for the players [12,13]. On the other hand, small-sided games (SSGs) are a particularly interesting approach due to the higher physiological acute responses achieved during this type of exercise, mainly when compared to the regular format of play [14]. The SSGs are smaller and adjusted formats of play, consisting of using a smaller number of players and changing the pitch configuration and some rules while keeping the dynamics of the real format of play [15,16]. SSGs have been extensively researched in the context of soccer training with different systematic reviews and meta-analyses providing interesting findings about the beneficial effects of these games on aerobic performance in comparison to other formats of training (e.g., running) [17,18,19]. Since beneficial effects in aerobic performance occur in soccer players, it could be interesting to identify whether such a fact might occur in untrained populations. Since small-sided games (SSGs) are smaller, these may become easier to use as recreational soccer with the purpose of promoting exercise among adult populations that love the game, by having a small group of friends to organize weekly practice [20]. Additionally, it is crucial to determine whether participating in SSGs can lower blood pressure among hypertensive patients. This is reasonable since this population seems to benefit from endurance training and adequate aerobic levels.

Although some systematic reviews have been done on the effects of recreational soccer in healthy and clinical populations [21,22], only one has summarized such findings related to SSGs [23]. This review [23] summarizes the findings related to different outcomes and populations (healthy and clinical) but does not include a meta-analysis. Additionally, as far as we know, specific reviews of recreational football conducted on clinical populations were specific to diabetes [24]. Due to the absence of a systematic review and the meta-analysis of the effects of soccer SSGs on untrained hypertensive population, this study aimed to assess the effects of SSG-based programs on the systolic and diastolic blood pressure of untrained hypertensive adults.

## 2. Materials and Methods

The Cochrane Collaboration guidelines were followed in this systematic review with meta-analysis [25]. The strategy of writing followed the guidelines of the Preferred Reporting Items for Systematic Reviews and Meta-analyses (PRISMA) [26]. The research question and eligibility criteria were defined using the Population, Intervention, Comparator, Outcomes, Study design (PICOS) approach (Table 1). The protocol of this systematic review was preliminary published in the INPLASY (International Platform of Registered Systematic Review and Meta-Analysis Protocols) with the protocol number INPLASY202090078 and the DOI code 10.37766/inplasy2020.9.0078.

### 2.1. Information Sources

The following electronic databases were used and searched for the current systematic review: Web of Science, Scopus, SPORTDiscus and PubMed. The search was conducted prior to the 29 September 2020, however, no limit to the publication date was defined. The search strategy defined the keywords and synonyms entries in these combinations: (“Soccer” OR “Football”) AND (“soccer training” OR “football training” OR “soccer game*” OR “conditioned game*” OR “small-sided soccer game*” OR “small-sided and conditioned game*” OR “SSG”) AND (“hypertension” OR “hypertensive” OR “blood pressure”).

### 2.2. Eligibility Criteria

The a priori inclusion criteria for this review were as follows: (i) randomized controlled trials including a control group and an intervention group exclusively using soccer SSGs; (ii) intervention and control groups (only passive) including an untrained hypertensive adult population; (iii) articles written in English; (iv) only full-text and original articles. *A posteriori* inclusion criteria were: (i) a measure of blood pressure (e.g., systolic blood pressure, diastolic blood pressure).

Studies were excluded on the basis that they: (i) included other sports than soccer; (ii) were not controlled study designs; (iii) combined interventions (SSGs and other training methods); and (iv) were review articles, letter or editorials, errata, invited commentaries or conference abstracts.

### 2.3. Extraction of Data

An Microsoft Excel sheet (Microsoft Corporation, Readmon, WA, USA) was designed and prepared to extract the data, assess the inclusion requirements and identify the selected articles by following the Cochrane Consumers and Communication Review Group’s data extraction template [27]. The registration and selection were independently performed by two authors (H.S., F.M.C.). A meeting between both authors occurred at the end of the process during which disagreement regarding study eligibility was resolved in a discussion with a third author (R.R.C.). The exclusion criteria for the articles were identified in the Excel sheet.

### 2.4. Data Items

The following outcomes were extracted from the included articles: (a) the diastolic blood pressure (mm Hg); and (b) systolic blood pressure (mm Hg). In addition to the outcomes, some information regarding the study characteristics were extracted, namely: (a) description of the participants (age, sex, number of participants); (b) information about the SSGs (format of play, pitch size); and (c) intervention details (training regimen, duration).

### 2.5. Assessment of Methodological Quality

The Physiotherapy Evidence Database (PEDro) scale was used to assess the methodological quality of the randomized controlled trials included in this systematic review and meta-analysis. The scale scores the internal study validity in a range from 0 (high risk of bias) to 10 (low risk of bias). Eleven items are measured in the scale. The criterion 1 is not included in the final score. Points for items 2 to 11 were only attributed when a criterion was clearly satisfied. The scale presents the following items and topics: N.º1: eligibility; N.º2: randomization of subjects; N.º3: allocation; N.º4: the groups were similar at baseline; N.º5: there was blinding of all subjects; N.º6: there was blinding of all therapists; N.º7: there was blinding of all assessors who measured at least one key outcome; N.º8: measures of at least one key outcome were obtained from more than 85% of the subjects initially allocated to groups; N.º9: all subjects for whom outcome measures were available received the treatment or control condition as allocated; No. 10: the results of between-group statistical comparisons were reported for at least one key outcome; and No. 11: the study provided both point measures and measures of variability for at least one key outcome.

The scoring process was made independently by two authors (F.M.C. and H.S.). In the final process, a meeting was organized in which comparisons were made and disagreements were solved in a discussion with the third author (R.R.C.). The agreement level between the two authors was analyzed using the Kappa correlation test, in which a level of k = 0.93 was obtained.

### 2.6. Summary Measures, Synthesis of Results, and Publication Bias

Pre-intervention and post-intervention means and standard deviations (SD) for the systolic and diastolic blood pressure measures (in SSGs and control groups) were used to calculate effect sizes (ES; Hedge’s *g*). The data were standardized using post-intervention SD values. Differences between studies that might impact the SSG-based effect were controlled using the random-effects model [28,29]. The ES values were shown with 95% confidence intervals (CI). The following thresholds were used to interpret the ES [30]: <0.2, trivial; 0.2–0.6, small; >0.6–1.2, moderate; >1.2–2.0, large; >2.0–4.0, very large; >4.0, extremely large. The *I*^2^ statistic was used to determine the heterogeneity level considering the following thresholds [31]: <25%, 25%–75%, and >75% considered to represent the low, moderate, and high levels of heterogeneity, respectively. The extended Egger’s test [32] was used to determine the risk of bias. In the case of bias, the trim and fill method was applied [33]. The statistical analysis was performed using the Comprehensive Meta-Analysis software (version 2; Biostat, Englewood, NJ, USA) in which a statistical significance was set at *p* ≤ 0.05.

## 3. Results

### 3.1. Study Identification and Selection

**Figure 1:**
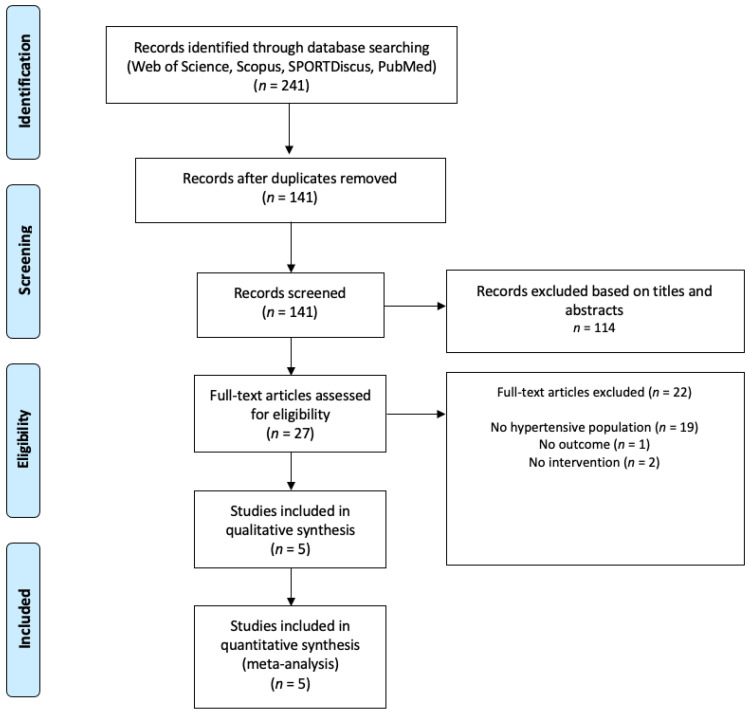
Preferred Reporting Items for Systematic Reviews and Meta-analyses (PRISMA) flow diagram highlighting the selection process for the studies included in the current systematic review.

An initial search revealed 241 titles. Those titles were organized in a reference manager software (EndNote^TM^ X9, Clarivate Analytics, Philadelphia, PA, USA). Duplicates (100 references) and were automatically or manually removed. Figure 1 shows the flow diagram for the number of articles in each step of the selection process. The five studies included provided the mean and standard deviation post-intervention data for the two main outcomes.

### 3.2. Study Characteristics

The characteristics of the five studies included in the meta-analysis can be found in Table 2.

Additionally, the details of the SSG-based programs can be found in Table 3. The included randomized controlled studies involved five individual experimental groups and 88 participants, and 68 participants in the five control groups.

### 3.3. Methodological Quality

The five included studies obtained seven points, thus suggesting “high” methodological quality (Table 4).

### 3.4. SSG vs. Control on Systolic Blood Pressure

A summary of the included studies and the results of systolic blood pressure reported before and after SSG-based intervention and control groups are provided in Table 5.

**Figure 2:**
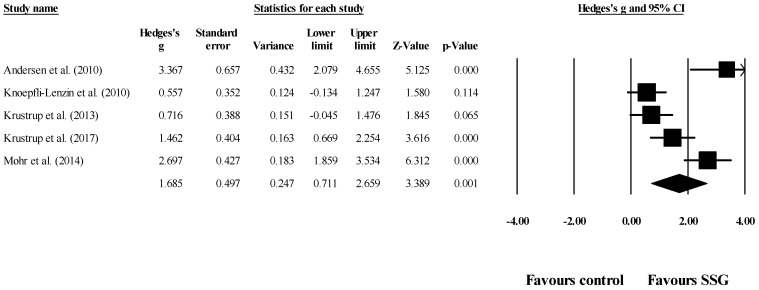
Forest plot of the changes in systolic blood pressure after participating in small-sided games (SSGs) compared to the control condition. Values shown are effect sizes (Hedges’s g) with 95% confidence intervals (CI). The size of the plotted squares reflects the statistical weight of each study.

Five studies provided data for systolic blood pressure, involving five experimental and five control groups (pooled *n* = 156). Results showed a large effect of SSG on systolic blood pressure (ES = 1.69; 95% CI = 0.71 to 2.66; *p* = 0.001; *I*^2^ = 85.2%; Egger’s test *p* = 0.101; Figure 2). The weight of each study in the analysis varied between 16.9% to 21.3%. The relative (%) reduction in systolic blood pressure among the included studies after SSG compared to the control was between −1.5% to −7.9%.

### 3.5. SSG vs. Control on Diastolic Blood Pressure

A summary of the included studies and results of diastolic blood pressure reported before and after the SSG-based intervention and control groups are provided in Table 6.

**Figure 3:**
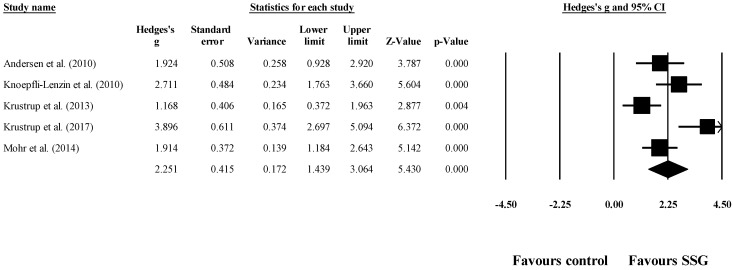
Forest plot of the changes in diastolic blood pressure after participating in small-sided games (SSGs) compared to the control condition. Values shown are effect sizes (Hedges’s g) with 95% confidence intervals (CI). The size of the plotted squares reflects the statistical weight of each study.

Five studies provided data for diastolic blood pressure, involving five experimental and five control groups (pooled *n* = 156). The results showed a very large effect of SSG on diastolic blood pressure (ES = 2.25; 95% CI = 1.44 to 3.06; *p* < 0.001; *I^2^* = 74.8%; Egger’s test *p* = 0.118; Figure 3). The weight of each study in the analysis varied between 17.1% and 22.3%. The relative (%) reduction in diastolic blood pressure among the included studies after SSG compared to the control was between −4.5% and −9.4%.

## 4. Discussion

People with higher fitness levels have a lower chance of developing or attenuating the progression of hypertension [39]. Therefore, exercise should be considered one of the main non-pharmacological interventions for helping hypertensive people [40]. Following this idea, this systematic review and meta-analysis tested the effects of recreational soccer SSGs on the blood pressure of men and women with hypertension. The findings of this study revealed a large and significant beneficial effect of SSG-based programs on the systolic and diastolic blood pressure of hypertensive adults when compared to control groups.

Considering the effects on systolic blood pressure, individual reports showed decreases of between 5.0% [37] and 8.8% [35] among SSG-based intervention groups, while changes in control groups varied between −0.7% [38] and −7.4% [35]. Among the five included randomized controlled trials, four of them [34,36,37,38] revealed a significant beneficial effect of SSG-based interventions when compared to control groups on systolic blood pressure. Overall, the magnitude of changes in systolic blood pressure was largely and significantly beneficial to those who participated in SSG-based interventions.

In the case of diastolic blood pressure, the reductions ranged between 4.6% [37] and 12.6% [35] in the intervention groups; meanwhile, in the case of controls, changes varied between −4.6% [35] and +4.8% [37]. The five included studies revealed significant beneficial effects of SSG-based programs when compared to control conditions. Overall, the changes in diastolic blood pressure were significantly beneficial to those who participated in SSG-based interventions.

Interestingly, the beneficial effects of SSG-based programs were the same regardless of sex, the period of intervention, or type of SSG formats at play. From the five included studies, three were conducted in men [34,35,36] and two in women [37,38]. Independent of the sex, diastolic blood pressure was significantly improved by the SSG-based interventions. No significant changes between the systolic blood pressure of intervention and control groups were observed in only one study [35] conducted in men (which had the shortest intervention period of 12 weeks). Overall, the beneficial results in both men and women are very promising since women commonly reduce their blood pressure more than men in certain conditions [41].

Considering the spectrum of interventions included in this systematic review and meta-analysis, the minimal period was three months [34], and the maximal period was 12 months [37]. The highest number of sessions a week (*n* = 3) occurred in programs with short intervention periods (12 and 15 weeks) [35,38]. In the remaining cases, the frequency was of two training sessions a week [34,36,37]. The formats of play implemented varied between 4 vs. 4 and 10 vs. 10. In three studies, the internal load during the games was registered, and the mean values were above 80% of maximal heart rate [35,36,38]. Since the programs presented similar acute effects with the type of aerobic exercise, it can be speculated that the positive effects follow the previous findings, suggesting that aerobic exercise is the most effective approach for reducing hypertension [42].

One of the limitations of the current systematic review and meta-analysis was that it was impossible (due to the limited number of included studies) to conduct a sub-group analysis to compare the effects of SSG-based interventions in men and women. Such a comparison should be considered in future original research to increase the understanding of the effects within these two populations. Other interesting sub-group analyses would involve investigating the medium- to long-term effects, the number of training sessions per week, or the type of SSGs employed. All of these analyses could not be conducted currently because of the reduced number of original studies included.

As a limitation of the original studies included in this meta-analysis, it needs to highlight the absence of information about monitoring the regular physical activity levels besides the exercise implemented (how changes occur in daily lives), as well as interactions with diet. Moreover, there was no specific consideration about the independent effect of exercise intensity and age on the changes occurring in the participants.

Future research should compare the effects of SSG-based interventions on different hypertension levels and identify the participants’ sensitivity to different levels of exercise. Moreover, a dose–response relationship can be conducted by monitoring the internal and external load performed by hypertensive adults during the exercise. Additionally, interactions between lifestyle patterns (e.g., dietary, physical activity, stress levels) and exercise should be considered to provide a better idea of how these factors act concurrently to explain changes in blood pressure. Finally, future research should give special consideration to responders and non-responders when reporting results.

The main practical applications of this systematic review and meta-analysis suggest that SSG-based programs conducted in an untrained hypertensive population can be used as a possible effective non-pharmacological intervention. Two to three weekly sessions of 50–60 min (total volume) of 4 vs. 4 to 8 vs. 8 formats of play are recommended.

## 5. Conclusions

This systematic review and meta-analysis revealed large and significant beneficial effects of SSG-based interventions on systolic and diastolic blood pressure among untrained hypertensive patients. Beneficial effects were found in both sexes, though a sub-group analysis was not possible. However, it is important to highlight that the I^2^ was higher than 50%, thus suggesting a high level of heterogeneity. The promising findings of this study support the idea that recreational soccer SSGs can act as a non-pharmacological intervention for people with hypertension, although, some cautions should be considered before a strong generalization of the findings.

## Figures and Tables

**Table 1:** Population, Intervention, Comparator, Outcomes, Study design (PICOS) approach.

PICOS Components	Details
Population	Untrained hypertensive adult populations of both sexes
Intervention	Small-sided games (SSG)-based training programs
Comparator	Passive controls
Outcomes	Blood pressure, systolic blood pressure, diastolic blood pressure
Study design	Randomized controlled trials and controlled trials

**Table 2:** Characteristics of the included studies and outcomes extracted.

Study	Mean Age (y)	N	Sex	Training Level	Design	CG	Protocol of Test Used in the Original Studies	Measure Extracted from the Tests in the Original Studies
Andersen et al. [34]	SSG: 46.7 ± 2.0 CG: 47.8 ± 1.8	SSG: 13 CG: 9	M	Untrained	RCT	Only received physician-guided traditional recommendation on cardiovascular risk factor modification	Supine position followed by 15 min rest. Blood pressure was recorded five times in both upper arms. Average values of 10 measurements were presented.	Systolic blood pressure (mm Hg) Diastolic blood pressure (mm Hg)
Knoepfli-Lenzin et al. [35]	SSG: 37.0 ± 4.0 CG: 38.0 ± 5.0	SSG: 15 CG: 17	M	Untrained	RCT	Continued their sedentary lifestyle	Measured in a sitting position	Systolic blood pressure (mm Hg) Diastolic blood pressure (mm Hg)
Krustrup et al. [36]	46 (range 31–54)	SSG: 20 CG: 10	M	Untrained	RCT	Only received physician-guided traditional recommendation on cardiovascular risk factor modification	Supine position followed by 15 min rest. Blood pressure was recorded five times in both upper arms. Average values of 10 measurements were presented.	Systolic blood pressure (mm Hg) Diastolic blood pressure (mm Hg)
Krustrup et al. [37]	SSG: 45.0 ± 6.0 CG: 45.0 ± 4.0	SSG: 19 CG: 12	W	Untrained	RCT	Continued their sedentary lifestyle	Supine position followed by 15 min rest. Blood pressure was recorded five times in both upper arms. Average values of 10 measurements were presented.	Systolic blood pressure (mm Hg) Diastolic blood pressure (mm Hg)
Mohr et al. [38]	SSG: 45.0 ± 3.0 CG: 43.0 ± 3.0	SSG: 21 CG: 20	W	Untrained	RCT	Continued their sedentary lifestyle	Measured after resting in supine position for 2 h. The average of 4 measurements was used.	Systolic blood pressure (mm Hg) Diastolic blood pressure (mm Hg)

N: sample size; RCT: randomized controlled trial; CG: control group; SSG: small-sided game group; M: men; W: women.

**Table 3:** Characteristics of SSG-based programs in the included studies.

Study	Duration (M/W)	d/w	Session/Person Per Week (n)	Total Sessions	SSG Formats	SSG Pitch Dimension (Length × Width)	SSG Area Per Player (m^2^)	Sets	Reps	Recovery between Sets (min)	Recovery between Sets (Intensity)	Total Work Duration (min)	Work Duration Per Set (min)	Work Intensity
Andersen et al. [34]	3 M	2	1.7 ± 0.2	22	5 vs.5 6 vs.6 7 vs.7	45–60 × 30–45 m	ND	2	-	5	ND	50	25	ND
Knoepfli-Lenzin et al. [35]	12 W	3	2.4 ± 0.2	ND	3 vs.3 4 vs.4 5 vs.5	ND	ND	ND	ND	ND	ND	50	ND	79.9 ± 4.5% HRmax
Krustrup et al. [36]	6 M	2	1.7 ± 0.5	43	5 vs.5 6 vs.6 7 vs.7	45–60 × 30–45 m	ND	4	ND	2	-	48	12	85.0 ± 7.0% HRmax
Krustrup et al. [37]	12 M	2	2.5 ± 0.4	128 ± 29	4 vs.4 to 8 vs.8	ND	ND	4	ND	2	-	48	12	ND
Mohr et al. [38]	15 W	3	3.0 ± 0.1	45	4 vs.4 to 10 vs.10	ND	ND	ND	ND	ND	ND	~60	ND	80.5 ± 1.1 to 98.9 ± 1.4 HRmax

SSGs: small-sided games; M: months; W: weeks; d/w: days per week; NR: not reported; m: meters; s: seconds; min: minutes; HRmax: maximal heart rate; ND: not described.

**Table 4:** Physiotherapy Evidence Database (PEDro) scale ratings.

Study	N.1 *	N.2	N.3	N.4	N.5	N.6	N.7	N.8	N.9	N.10	N.11	Total **
Andersen et al. [34]	1	1	1	1	0	0	0	1	1	1	1	7
Knoepfli-Lenzin et al. [35]	1	1	1	1	0	0	0	1	1	1	1	7
Krustrup et al. [36]	1	1	1	1	0	0	0	1	1	1	1	7
Krustrup et al. [37]	1	1	1	1	0	0	0	1	1	1	1	7
Mohr et al. [38]	1	1	1	1	0	0	0	1	1	1	1	7

*: PEDRro scale items number. **: the total number of points from a possible maximal of 10.

**Table 5:** Summary of the included studies and results of systolic blood pressure before and after intervention.

Study	Group	N	Before Mean ± SD	After Mean ± SD	After–Before (Δ%)
Andersen et al. [34]	SSG	13	150 ± 3	138 ± 2	−8.0
Knoepfli-Lenzin et al. [35]	SSG	15	136 ± 3	124 ± 4	−8.8
Krustrup et al. [36]	SSG	20	151 ± 10	139 ± 9	−7.9
Krustrup et al. [37]	SSG	19	140 ± 1	133 ± 3	−5.0
Mohr et al. [38]	SSG	21	139 ± 3	127 ± 4	−8.6
Andersen et al. [34]	CG	9	153 ± 3	148 ± 2	−3.3
Knoepfli-Lenzin et al. [35]	CG	17	136 ± 3	126 ± 3	−7.4
Krustrup et al. [36]	CG	10	152 ± 7	146 ± 6	−3.9
Krustrup et al. [37]	CG	12	135 ± 9	132 ± 2	−2.2
Mohr et al. [38]	CG	20	134 ± 4	133 ± 4	−0.7

SSG: small-sided game based-program; CG: control group.

**Table 6:** Summary of the included studies and results of diastolic blood pressure before and after intervention.

Study	Group	N	Before Mean ± SD	After Mean ± SD	After–Before (Δ%)
Andersen et al. [34]	SSG	13	91 ± 2	84 ± 2	−7.7
Knoepfli-Lenzin et al. [35]	SSG	15	87 ± 3	76 ± 3	−12.6
Krustrup et al. [36]	SSG	20	92 ± 7	84 ± 5	−8.7
Krustrup et al. [37]	SSG	19	87 ± 9	83 ± 2	−4.6
Mohr et al. [38]	SSG	21	86 ± 2	80 ± 3	−7.0
Andersen et al. [34]	CG	9	95 ± 2	92 ± 2	−3.2
Knoepfli-Lenzin et al. [35]	CG	17	87 ± 2	83 ± 2	−4.6
Krustrup et al. [36]	CG	10	96 ± 6	94 ± 5	−2.1
Krustrup et al. [37]	CG	12	83 ± 5	87 ± 2	4.8
Mohr et al. [38]	CG	20	82 ± 3	81 ± 2	−1.2

SSG: small-sided game based-program; CG: control group.
